# Fish Innate Immune Response to Viral Infection—An Overview of Five Major Antiviral Genes

**DOI:** 10.3390/v14071546

**Published:** 2022-07-15

**Authors:** Maria del Mar Ortega-Villaizan, Veronica Chico, Luis Perez

**Affiliations:** Instituto de Investigación Desarrollo e Innovación en Biotecnología Sanitaria de Elche (IDiBE—UMH), 03202 Elche, Spain; mortega-villaizan@umh.es (M.d.M.O.-V.); vchico@umh.es (V.C.)

**Keywords:** viral disease, fish, innate immunity, interferon, mx, cyprinids, salmonids

## Abstract

Fish viral diseases represent a constant threat to aquaculture production. Thus, a better understanding of the cellular mechanisms involved in establishing an antiviral state associated with protection against virus replication and pathogenesis is paramount for a sustainable aquaculture industry. This review summarizes the current state of knowledge on five selected host innate immune-related genes in response to the most relevant viral pathogens in fish farming. Viruses have been classified as ssRNA, dsRNA, and dsDNA according to their genomes, in order to shed light on what those viruses may share in common and what response may be virus-specific, both in vitro (cell culture) as well as in vivo. Special emphasis has been put on trying to identify markers of resistance to viral pathogenesis. That is, those genes more often associated with protection against viral disease, a key issue bearing in mind potential applications into the aquaculture industry.

## 1. Introduction

Following virus challenge, fish mount an early antiviral response by activating the innate immune system [1,2,3] and later on another response takes place in the way of antibody production [4,5] and cytotoxic T-cell immunity [6]. Here, the focus will be set on the activation of five genes in the interferon pathway that could be regarded as the most relevant in the response to viral infection in fish. The five selected genes are the most widely reported virus-induced genes associated with antiviral activity in fish: type I interferon (*ifn*) [7] the myxovirus resistant protein (*mx*) [8], the interferon stimulated gene 15 (*isg15*) [9], viperin (also named *vig*, *rsda2*) [10], and the grass carp reovirus induced gene (*gig*) [11].

Interferons (IFN) are secreted proteins in the range of 20–23 KDa. After binding to a receptor on the cell membrane, they activate a number of cellular pathways that ultimately lead to the transcriptional activation of the interferon-stimulated genes (ISGs) [7]. Two types of interferons have been found in teleost fish: type I and type II [12,13]. In this work, the term interferon (*ifn*) will refer to type I interferons (IFNα and IFNβ).

Mx proteins (60–70 KDa) belong to the superfamily of interferon-induced GTPases. Mx genes are highly polymorphic, with three Mx isoforms (Mx1, Mx2, Mx3) in salmonids and perciforms, and up to seven isoforms (MxA to MxG) in zebrafish [8]. ISG15 is a highly conserved ubiquitin-like protein (15 KDa) with antiviral activity [9,14]. Originally reported as a VHSV-induced gene (*vig*) in rainbow trout leukocytes [15,16], Viperin (42 KDa) is one ISG with direct antiviral activity [10]. Unlike the other four antiviral genes that are present in all vertebrates, Gig proteins are specific for fish. They display good antiviral activity and may be induced both in an *ifn*-dependent as well as an *ifn*-independent way [11].

There is an extensive body of literature on the activation of the innate immune response following viral infection of fish. A fair number of reviews have covered this subject, focusing either on one specific virus or one fish species [17,18,19]. A large portion of the data available on fish immune response against viral infection has been drawn from studies conducted on cyprinid and salmonid fish species. This is not surprising, since carp and salmon are arguably amongst the most widely cultivated fish species globally [20]. Thus, specific sections dealing with those two groups of fish will be presented, while other fish will be discussed together in a separate section. “Other fish” will mainly refer to orders Perciformes (i.e., sea bass, sea bream, tilapia) and Pleuronectiformes (flat fish species).

## 2. ssRNA Viruses

Viruses with single-stranded RNA (ssRNA) genomes comprise the largest number of viruses reported to cause disease in farmed fish, including long time known pathogens of carp (spring viremia of carp virus, SVCV) and pathogens affecting salmon such as salmon infectious hematopoietic necrosis virus (IHNV), infectious salmon anemia virus (ISAV), salmonid alphavirus (SAV), and viral hemorrhagic septicemia virus (VHSV) (Table 1).

### 2.1. ssRNA Virus Infection of Cyprinid Fish

Rhabdoviruses are arguably one of the viral families with greater impact on farmed fish. With respect to cyprinid species, SVCV is the most widely spread viral pathogen [21]. SVCV has been shown to be a powerful inducer of *ifn* expression in carp cells [22,23]. Likewise, the up-regulation of *ifn* gene expression by SVCV infection has been found in zebrafish cells [24,25,26]. Also in SVCV-infected zebrafish cells, *mx* gene transcription appears to be activated as early as 1hpi [27], suggesting an interferon-independent stimulation of *mx* in this case. In some instances, even an abortive infection with a fish rhabdovirus can lead to higher *ifn* and *mx* expression in cultured cells [28]. The stimulation of the *vig/viperin* gene expression has been reported in SVCV-infected cells [29,30,31,32]. SVCV infection has been also shown to up-regulate the transcription of the *gig* gene and the synthesis of GIG protein in zebrafish cells [33].

Along with the in vitro studies on cell culture, the in vivo interferon response of fish to rhabdoviruses has been largely examined in a number of cyprinid fish species. Increased levels of *ifn* and *mx* gene expression have been reported in carp and goldfish infected with SVCV, not only in internal organs, but also in skin [34,35,36,37].

Although not an aquacultured species, zebrafish (also a cyprinid fish) has been an extensively employed experimental model to investigate innate immune response to rhabdoviruses [38,39,40]. Overall, there is ample evidence of SVCV being a powerful inducer of all five genes (*ifn*, *mx*, *isg15*, *vig/viperin*, and *gig*) in zebrafish and carp [24,26,32,41,42,43]. Interestingly, in SVCV-infected zebrafish, some isoforms of the *mx* gene had their expression increased while other isoforms seemed to be down-regulated [25,44]. In contrast, none of the four zebrafish *ifn* genes were induced in larvae challenged with IHNV [45], which is suggestive of significant differences in the innate immune response between the larvae and adult state in fish. In fact, infections with IHNV and SVCV do trigger *ifn* expression in adult zebrafish [46]. Moreover, the levels of *mx* expression remain high in zebrafish that survived a VHSV challenge [38]. The stimulation of *mx* gene expression appeared particularly high in the gills [27] and liver [40] of the infected fish. *Ifn* expression was high in the kidney, whereas *vig/viperin* gene expression was found up-regulated in every tissue [47].

### 2.2. ssRNA Virus Infection of Salmonid Fish

SsRNA viruses are good inducers of *ifn* response in salmon cells [48]. Regarding the timing of gene expression, interferon-stimulated genes would be expected to be up-regulated after interferon induction, but that does not seem to be always the case. For instance, in salmon cells infected with ISAV, a peak of *isg15* at 24 hpi is followed by high *ifn* expression at 48 hpi [49]. In contrast, a delayed (5dpi) up-regulation of both *ifn* and *mx* genes in SAV-infected salmon cells have been reported [50]. 

Infection with a virulent strain of VHSV provokes the transcriptional activation of *ifn* and *mx* in rainbow trout [51]. The ex-vivo transfection of rainbow trout red blood cells (RBCs) with a G-VHSV plasmid DNA vaccine has been reported to trigger *mx* expression [52]. The up-regulation of *mx* gene expression (with up to 1700-fold increment in liver) has been reported in salmonid fish infected by IHNV [53,54,55,56], as well as in salmon displaying clinical signs but not experiencing mortality [57]. ISAV infection of salmon also leads to increasing *ifn* and *mx* levels, albeit at later times (not until 6–8 days post challenge) [58,59]. This may be one of the cases where the innate response would be more a consequence of the ongoing viral replication rather than an early antiviral response of the fish, something that has been also suggested in IHNV-infected salmon [55]. Similar to viral infections of carp, the up-regulation of *gig* or gig-like gene expression has been reported in ISAV-infected salmon [60]. The transcriptional activation of *gig* along with that of *mx* and *vig/viperin* has been observed in salmon between 2 and 4 weeks after challenge with SAV [61,62,63].

### 2.3. ssRNA Virus Infection of Other Fish

Many species of fish other than cyprinid and salmonid fish are known to be susceptible to ssRNA virus infections, also displaying a subsequent activation of the interferon pathway. In Pleuronectiformes (flatfish species), a potent induction of *ifn* and *mx* gene expression have been detected after challenge with rhabdoviruses HIRRV (hirame novirhabdovirus) and VHSV [64,65,66,67,68], with up to 16.000-fold increment of *ifn* levels at day 3 after VHSV infection. Likewise, high RNA expression levels of *mx* has been measured in the kidney and spleen of VHSV-infected perch [69].

Nodaviruses pose a major threat to cultured marine fish worldwide, particularly perciforms species. Nervous necrosis virus (NNV)-infected seabass displays strong *ifn*, *mx*, and *isg15* responses in 6–12 h both in the kidney and brain [70,71]. The up-regulation of *mx* has been reported in barramundi brain cells persistently infected with nodavirus [72,73]. On this regard, the capsid protein of nodaviruses has been pointed out to be a strong inducer of innate immune genes transcription [74]. 

Both *ifn* and *mx* responses have been detected in tilapia lake virus (TiLV)-infected tilapia and zebrafish larvae [75,76].

## 3. dsRNA Viruses and Poly I:C

The Birnaviridae and Reoviridae families of viruses with double-stranded RNA (dsRNA) genomes include two very important fish pathogens such as grass carp reovirus (GCRV) and IPNV (Table 1). DsRNA has been recognized as one molecular pattern sensed as “strange” by the cell. Thus, dsRNA is regarded as a very effective inducer of the innate immune cell response. In particular, the synthetic dsRNA polyinosinic-polycytidylic acid (poly I:C) has been so widely used to study the cell response to dsRNA that it deserves an inclusion in this section along the response to actual dsRNA viruses.

### 3.1. dsRNA Virus Infection and polyI:C in Cyprinid Fish 

Poly I:C consistently induces a rapid and strong interferon response in carp and zebrafish cells [22,23,25,26,29,77]. *Mx* RNA levels increased significantly following *epithelioma papulosum cyprini* (EPC) cells treatment either with poly I:C or with conditioned medium from cells treated with poly I:C [78]. As proof that it is a good virus mimic, poly I:C has been shown to be an effective inducer of *gig* and *vig*/*viperin* gene expression in vitro [10,33,79]. It is worth mentioning that the *gig* gene was discovered precisely in reovirus-infected cells [11].

The infection of zebrafish cells with GCRV led to an increase in *mx* gene expression levels over 100-fold [27]. Notably, fish reoviruses have the remarkable capacity of inducing interferon and interferon-mediated response even when the viral particle has been inactivated [7,11,80,81], which is suggestive that the input of dsRNA may be sufficient to trigger the cell response without the need of viral replication. High transcription levels of *mx* that correlated with the production of interferon-like activity in supernatants of birnavirus-infected cyprinid fish cells has also been reported [82,83].

In vivo, poly I:C has been most frequently delivered to fish by intraperitoneal (i.p.) injection. The transcriptional activation of *ifn* and *mx* genes in head kidney following i.p. injection of poly I:C has been determined in a zebrafish model [84] with a peak of expression after 48 h [85]. Intramuscular injection has also been proven effective in triggering a host response to poly I:C in cyprinid fish [86].

GCRV infection elicits a transcriptional activation of *mx* and *isg15* in the spleen and kidney of carp, with the more virulent strains being the more potent inducers [87,88]. Following GCRV infection of goldfish, *ifn* RNA levels were particularly high in the skin [37]. This finding would be an indication that epithelial tissues may have been overlooked in the study of the immune response in fish.

### 3.2. dsRNA Virus Infection and polyI:C inSalmonid Fish 

Many viruses of salmonid fish have been shown to be inhibited in some degree by the treatment (usually prior to infection) of the cells with poly I:C. Depending on the particular cell line, cells can be simply exposed to the synthetic dsRNA, whereas in some other cases a transfection reagent was required for poly I:C to enter the cells and exert the antiviral effect. The latter is the case of the rainbow trout gill (RTgill) cell line where *ifn* gene expression was significantly increased after poly I:C transfection [89]. In salmon, chinook salmon embryo cells (CHSE-214) cells transfection is also required for poly I:C to trigger *ifn*, *mx* and *vig/viperin* transcription [90,91]. In other salmonid cell lines, simple exposure to poly I:C is sufficient to induce *mx* gene expression [92]. Taken together, these studies support the notion that a robust *ifn/mx* response would be the main factor underlying the antiviral state induced by poly I:C. 

Some authors have investigated the molecular weight-dependent activity of poly I:C, finding that high molecular weight (HMW) poly I:C is a stronger inducer of *mx* than the low molecular weight (LMW) poly I:C in rainbow trout gonad (RTG-2) cells [93]. This was somehow expected since the HMW poly I:C is closer to the size of the viral dsRNA genomes. Indeed, viral dsRNA isolated from reovirus-infected cells has been proven an effective inducer of interferon [94].

Further support for the poly I:C treatment accurately mimicking viral infection in vitro, the birnavirus IPNV has been proven to be good inducer of antiviral genes (*ifn* and *mx*) in cells where IPNV can establish a productive infection [56,95]. Likewise, transcriptional activation of *mx* has been observed in rainbow trout cells infected with chum salmon reovirus (CSV) [96]. Interestingly, in the abortive infection of rainbow trout RBCs with IPNV, still *ifn* and *mx* gene expression was found up-regulated [97].

Considering the strong antiviral effect of poly I:C in cell culture, it is not surprising to find in the literature a number of attempts to prove the antiviral activity of poly I:C in the whole fish, with reports of successful application of poly I:C in VHSV-challenged fish [84,98]. It is worth mentioning that previous poly I:C inoculation resulted in higher anti-VHSV antibody production in flounder [98].

In piscine reovirus (PRV)-infected salmon, higher levels of *ifn*, *mx*, *isg15*, and *vig/viperin* genes expression have been determined [57,99]. PRV is one virus capable of establishing productive infection in RBCs where it triggers a rapid (as early as 1dpi) *ifn* response followed by a peak of *mx* expression [100].

The birnavirus IPNV has been a classic model of dsRNA virus infection in salmonids. Several authors have demonstrated that the RNA levels of *ifn*, *mx*, and *gig* genes were increased in salmon fish challenged with IPNV [59,101,102,103].

The less studied piscine myocarditis virus (PMCV) also induces a response in infected salmon that is highlighted by the overexpression of the antiviral gene *vig/viperin* [104].

### 3.3. dsRNA Virus Infection and poly I:C in Other Fish 

Other groups of teleost fish respond to poly I:C stimulation in a similar way to that of cyprinid and salmonid fish. Catfish cell lines exhibit a rapid (in only 6 h after stimulation) up-regulation of *ifn*, *mx*, and *isg15* after exposure to poly I:C both in vitro and in vivo [105,106]. In vivo, i.p. injection of poly I:C also correlates with increased *mx* and *isg15* levels in rock bream and large yellow croaker [107,108]. 

Regarding the response to a dsRNA virus challenge, bluegill (*Lepomis machrochirus*) cells infected with infectious pancreatic necrosis virus (IPNV) show increased levels of *ifn* and *mx* from early times of infection [95]. 

There is a limited number of studies on fish other than cyprinids and salmonids. In one rare example on IPNV infection in cod causing death, the induction of *isg15* following IPNV challenge has been reported [9]. In birnavirus-infected flounder, *mx* was overexpressed at the transcriptional level [109].

## 4. DNA Viruses

Like ssRNA and dsRNA, a pathogen–derived dsDNA can be sensed as “strange” by cell pattern recognition receptors and mount a reaction to that exogenous molecule [110]. The two major groups of fish DNA viruses are the Iridoviridae and Herpesviridae families.

The iridovirus LCDV (lymphocystis disease virus) activates *mx* gene expression in Senegalese sole [111] as well as other cytokines. High Mx protein levels in RBIV-infected turbot have been also reported [112]. In contrast, neither *ifn* nor *mx* mRNA levels were increased in sea bream infected with LCDV [113]. The activation of *mx* transcription has also been observed in mandarin fish challenged with infectious spleen and kidney necrosis virus (ISKNV) [114].

In herpesvirus-infected carp, the interferon response shows a good correlation with viral loads [47,115]. In any case, the innate immune response of fish to herpesviruses seems to be variable. While *ifn* expression levels did not show significant differences in cyprinid herpesvirus (CyHV)-infected carp [116], stimulation of *mx* transcription did occur in catfish infected with channel catfish virus (CCV) [106].

## 5. Comparative Summary of ssRNA, dsRNA and DNA Viruses

Molecular patterns associated to pathogens are recognized by the cell pattern recognition receptors. One group of receptors with a decisive role in sensing foreign ssRNA, dsRNA, or DNA are the toll-like receptors (TLRs). In fish as well as in other vertebrates, while TLR9 recognizes pathogen DNA, TLR3 is the receptor for foreign dsRNA, and both TLR7 and TLR8 would sense viral ssRNA [117]. 

Overall, from the available information in the literature, it can be concluded that ssRNA viruses and dsRNA viruses elicit a rather similar host innate immune response (Table S1). Discrepancies come up when comparing RNA and DNA viruses. Whereas the majority of the RNA viruses reviewed in the previous sections replicate in the cytoplasm, the life cycle of many DNA viruses requires the cell nucleus. Although there are exceptions to this principle (i.e., orthomyxoviruses such as ISAV with nuclear transcription and replication), it can be taken as a rule of thumb. Thus, for a given virus, having either a nuclear or a cytoplasmic life cycle may result in differences regarding the modulation of the innate immune response within the infected cell. The transcriptional activation of *vig/viperin* gene was first found only in RNA virus-infected fish, but later on the up-regulation of *vig/viperin* in iridovirus-infected fish was also reported [118,119]. Recently, microarray analysis has revealed the stimulation of *gig* in salmon infected with a poxvirus, in addition to *mx*, *isg15*, and *vig/viperin* [120]. 

As a general rule, RNA viruses tend to activate the transcription of a wider number of antiviral genes, while the response to DNA viruses seems restricted to fewer genes (Table 2). Four of the key innate immune genes (*ifn*, *mx*, *isg15*, *vig/viperin*) appear to be up-regulated by almost every RNA virus studied. In studies conducted with both types of viruses, differences between RNA and DNA viruses have been reported. For instance, in carp cells, the herpesvirus CyHV seems to be a poor inducer of interferon compared to SVCV [22]. This lack of IFN stimulation upon CyHV challenge has been verified in vivo [121]. 

With respect to poly I:C compared to viral RNA, some differences have been found as well regarding the cell response. In salmon TO cells, while poly I:C stimulation triggers first *ifn* gene expression followed hours later by an increase of *mx* RNA levels, ISAV infection resulted in a rapid overexpression of *mx* and *isg15* genes followed by a late induction of *ifn* gene expression [122]. In some instances, the differences are related to the specific isoform of the gene that is activated; in rainbow trout, while poly I:C stimulated *mx3* expression, VHSV induced *mx1* and *mx2* [123].

## 6. Correlation between Up-Regulation of Innate Immunity Genes and Protection from Viral Infection and Disease

As previously discussed, there is ample support for the activation of innate immune genes after viral challenge in farmed fish species. Now the key question is: Does it really matter? Does the interferon-mediated response guarantee protection against disease, limiting morbidity and ensuring fish survival? Let us evaluate the evidence.

One point that should be underscored in the first place: a robust interferon response does not necessarily mean survival to virus infection. SVCV is a potent inducer of interferon and Mx in carp cells, but the outcome after exposure to SVCV is the death of the infected cells nevertheless [22,124]. This situation has been confirmed in vivo, with high *ifn* and *mx* induction but also high mortality in SVCV-infected zebrafish [124,125]. The transcriptional induction of the *mx* gene is considered a hallmark of the antiviral response in fish. Challenge with a virulent strain of VHSV leads to high interferon and *mx* induction but also high mortality in infected rainbow trout [51]. Thus, in some virus-host situations, a potent *ifn* response would be more the result of increasing viral replication rather than an early mechanism of antiviral defense [55]. Salmonid fish cell lines such as RTG-2 have shown good responsiveness to VHSV infection. Interestingly, the low pathogenic strains of VHSV exhibit the higher stimulation capacity of *mx* gene expression, leading to the hypothesis that the highly pathogenic VHSV strains would be somehow able to restrict *mx* expression at least in vitro [126].

In contrast to viral infections in the whole fish, the transfection of cultured fish cells with plasmids encoding interferon or other interferon-stimulated antiviral genes has been usually successful in halting viral replication [14,36,80]. In one case, the inhibition was achieved by the overexpression of the antiviral gene *gig* in carp cells infected with the reovirus GCRV [80]. Conditioned medium from cells producing interferon has been in some instances capable of blocking viral (SVCV and GCRV) replication in cell culture [37,77], evidencing the synthesis and release of antiviral factors to the medium by the transfected cells.

Poly I:C has consistently demonstrated its capacity to protect (either completely or partially) cells from the virus-induced cytopathic effect. Examples of the antiviral activity of poly I:C are found in rainbow trout cells infected with VHSV [89,94,127], salmon cells infected with IPNV [90], and sea bream cells infected with birnavirus [128]. In contrast, poly I:C was not so successful in blocking nodavirus replication in grouper cells [129]. The same antiviral effect can be induced by in vitro synthesized dsRNA [79] or with isolated viral dsRNA [94]. The novel CRISPR/Cas genome-editing tool is currently assisting in the elucidation of the detailed mechanism underlying poly I:C induced antiviral effect, by knocking out candidate genes along the interferon pathway one by one [130,131,132].

The protective effect of poly I:C has been corroborated in vivo in zebrafish intraperitoneally injected with poly I:C and challenged with VHSV [85]. However, it appeared to be more a delay in virus-induced pathogenesis and mortality than actual protection [84]. In olive flounder, a drastic decrease of VHSV-induced mortality can be achieved by poly I:C administered intraperitoneally [133]. In a similar way, RBIV iridovirus replication in rock bream was inhibited by i.p. injection of poly I:C [107].

Intramuscular injection of turbot with a plasmid encoding interferon reduced the mortality caused by VHSV from 100% to 47%, in conjunction with a 200-fold up-regulation of *mx* gene expression in muscle [67]. Additionally, the high transcriptional levels of *mx* found in zebrafish survivors of a VHSV challenge may be associated with their high resistance to a second VHSV infection [38], although in this case the role of the adaptive immune response should not be neglected. In contrast, the injection of a *mx* gene encoding plasmid into turbot did not appear to induce any degree of protection against RIBV [112]. Correlation between higher interferon production and resistance to IPNV has been reported in Atlantic salmon [102]. However, the higher levels of *mx* and other interferon stimulated genes in IPNV-infected salmon may just be a result of the higher viral replication in susceptible fish [103]. Likewise, in SVCV-infected carp, a stronger interferon response was directly correlated with higher viral loads [47]. In contrast, zebrafish rag −/− mutants defective in adaptive immune response have been used to associate higher innate immune response with less susceptibility to SVCV infection [134].

Evidence in favor of the protective efficacy of the innate response has been found by analyzing the susceptibility to viral disease of selected populations of fish, where genetically resistant individuals seemed to exhibit a stronger innate immune response [135,136,137]. In some other instances, however, up-regulation of *ifn*, *mx*, and *vig/viperin* was associated with acute infection [55]. In tilapia infected with TiLV, higher *mx* response did not correlate with reduced infection [75]. 

Overall, the real protective effect of a strong *ifn*/*ifn*-induced gene response in vivo remains inconclusive, although there is a body of evidence pointing to the fact that a natural endogenous innate immune response would not suffice to stop virus spread in the organism.

## 7. Final Remarks

In the last decade, a large body of knowledge on the innate immune response in teleost fish has been built up by researchers on viral diseases of fish. The question is how the fish farming industry can benefit from all this available information.

As a starting point of thinking, viruses exist because they “learned” how to overcome the natural host immune response, reaching a situation where the production of interferon and other antiviral proteins become ineffective against virus replication within the host. However, that does not necessarily mean that viruses would prevail against a human- engineered, enhanced immune response elicited by a specific treatment or vaccine. Therefore, when thinking on prophylactic measures, we should not only focus on improving the capacity of producing neutralizing antibodies, but rather on triggering a rapid and robust antiviral state in the fish by means of the innate immune system. The experimental results reviewed in here indicate that the latter may be feasible. However, first there are some issues that will have to be sorted out. For instance, little information is available on the duration of the antiviral effect established through the interferon and related pathways. This is a key factor, since a long-lasting exacerbated innate response could be deleterious to the fish. Therefore, a novel way of thinking is that protection against disease in the long term might come from the more durable epigenetic changes in the host chromatin [41,137], resulting in a faster and enhanced response upon a second pathogen invasion.

Better understanding of the key responders to viral infection will boost the advancement of fish vaccinology. In that regard, the knowledge on some lesser-known areas such as mucosal immunity should be expanded. The use of omics techniques will contribute to provide an unbiased and comprehensive picture and may lead to the discovery of new players in the virus-host interplay. With such a multiplicity of factors playing a part in viral pathogenesis (e.g., species susceptibility, genetic variability among fish individuals, age/size of fish, water temperature, and virulence of viral isolates), establishing clear cut patterns is not going to be an easy task. This could be further complicated by the emergence of novel aquatic viruses under changing climatic conditions and increasing sea water temperatures in the coming years.

## Figures and Tables

**Table 1:** Viruses cited in this work.

ssRNA	Name	Acronym	Main host
Amnoonviridae	Tilapia lake virus	TiLV	Tilapia
Nodaviridae	Nervous necrosis virus	NNV	Perciformes
Orthomyxoviridae	Infectious salmon anemia virus	ISAV	Salmonid
Rhabdoviridae	Hirame novirhabdovirus	HIRRV	Salmonid
	Infectious hematopoietic necrosis virus	IHNV ^1^	Salmonid
	Spring viremia of carp virus	SVCV	Cyprinid
	Viral hemorrhagic septicemia virus	VHSV ^2^	Salmonid
Togaviridae	Salmonid alphavirus	SAV	Salmonid
**dsRNA**			
Birnaviridae	Infectious pancreatic necrosis virus	IPNV	Salmonid
Reoviridae	Chum salmon reovirus	CSV	Salmonid
	Grass carp reovirus	GCRV	Cyprinid
	Piscine reovirus	PRV	Salmonid
Totiviridae	Piscine myocarditis virus	PMCV	Salmonid
**dsDNA**			
Herpesviridae	Channel catfish virus (Ictalurid herpesvirus)	CCV	Ictalurid
	Cyprinid herpesvirus	CyHV	Cyprinid
Iridoviridae	Infectious spleen and kidney necrosis virus	ISKNV	*S. chuatsi*
	Lymphocystis disease virus	LCDV	Sea bream, sole
	Rock bream iridovirus	RBIV	*O. fasciatus*

^1^ Salmonid novirhabdovirus; ^2^ Piscine novirhabdovirus.

**Table 2:** Innate immune genes *ifn*, *mx*, *isg15*, *vig/viperin*, and *gig* stimulation by viruses of teleost fish.

**ssRNA**	**CYPRINIFORMES**	**SALMONIFORMES**	**OTHERS**
TiLV			*ifn*, *mx*
NNV	*mx*		*ifn*, *mx*, *isg15*
ISAV		*ifn*, *mx*, *isg15*, *gig*	
IHNV	*ifn*	*ifn*, *mx*, *vig*	
SVCV	*ifn*, *mx*, *vig*, *isg15*, *gig*		
VHSV	*ifn*, *mx*, *vig*	*ifn*, *mx*, *isg15*	*ifn*, *mx*
SAV		*ifn*, *mx*, *vig*, *gig*	
**dsRNA/polyIC**	**CYPRINIFORMES**	**SALMONIFORMES**	**OTHERS**
poly I:C	*ifn*, *mx*, *vig*, *gig*	*ifn*, *mx*, *vig*	*ifn*, *mx*, *isg15*
IPNV	*mx*	*ifn*, *mx*, *gig*	*ifn*, *mx*, *isg15*
CSV		*mx*	
GCRV	*ifn*, *mx*, *isg15*		
PRV		*ifn*, *mx*, *isg15*, *vig*	
PMCV		*vig*	
**dsDNA**	**CYPRINIFORMES**	**SALMONIFORMES**	**OTHERS**
CCV			*mx*
ISKNV			*mx*
LCDV			*mx*
RBIV			*mx, isg15, vig*

## Data Availability

Not applicable.

## Supplementary Materials

The following supporting information can be downloaded at: https://www.mdpi.com/article/10.3390/v14071546/s1, Table S1: In vivo stimulation of the antiviral genes reviewed in this work in the main species and groups of fish.
