# Supplementary material for: Fish Innate Immune Response to Viral Infection—An Overview of Five Major Antiviral Genes
Source: Viruses. 2022 Jul 15;14(7):1546. doi: 10.3390/v14071546 (PMC9317989; doi:10.3390/v14071546)
Supplement: Supplementary file 1 [file viruses-14-01546-s001.zip › viruses-1773568-SI.pdf]

Table S1. In vivo stimulation of the antiviral genes reviewed in this work in the main species and groups of fish. (Numbers are the reference numbers in the Bibliography).

		ifn	*mx*	*vig*	*isg15*	*gig*
**ssRNA viruses**	Carp	34,35,36,43, 47	43	47	43	
	Zebrafish	24,25,26,40, 42,46	25,27,32,38, 40	32	32	41
	Rainbow trout	51	51,52,54,56, 123			
	Salmon	59	53,55,57,58, 61	63	63	60,63
	Perciforms	70,71,75,76	69,70,71,72, 73,75,76	70		
	Pleuronectiforms		64,66			
**Poly I:C dsRNA viruses**	Carp		88		87	
	Zebrafish	84,85				
	Rainbow trout		123			
	Salmon	99,100,102	57,59,99,100 101	99,104	99	
	Perciforms	95	95			
	Pleuronectiforms		109			
**DNA viruses**	Carp	115				
	Perciforms		114			
	Pleuronectiforms		111,112			
